# Innovative Web-Based Future Planning and Well-Being for Caregivers of Individuals With Intellectual and Developmental Disabilities: Protocol of a Pragmatic Randomized Controlled Trial

**DOI:** 10.2196/77184

**Published:** 2025-10-01

**Authors:** Caren Steinway, Charmaine Wright, Silvia Kwak, Olivia Teng, Abigail Seide, John Berens, Jason Woodward, Ilka Riddle, Thomas Davis, Adam Greenberg, Dava Szalda, Justine Shults, Jane Cerise, Sophia Jan

**Affiliations:** 1 Northwell Health New Hyde Park, NY United States; 2 Department of Pediatrics Zucker School of Medicine at Hofstra Hempstead, NY United States; 3 Department of Medicine Christiana Care Health System Wilmington, DE United States; 4 Department of Medicine Baylor College of Medicine Houston United States; 5 Department of Pediatrics Indiana University School of Medicine Indianapolis, IN United States; 6 Division of Developmental and Behavioral Pediatrics Cincinnati Children's Hospital Medical Center Cincinnati, OH United States; 7 Department of Medicine Geisinger Medical Center Danville, PA United States; 8 Department of Pediatrics Children's Hospital of Philadelphia Philadelphia, PA United States; 9 Department of Medicine University of Pennsylvania Philadelphia, NY United States; 10 Department of Biostatistics, Epidemiology and Informatics Perelman School of Medicine at University of Pennsylvania Philadelphia, PA United States

**Keywords:** innovative long-term care planning tool, intellectual and developmental disabilities, caregivers, health disparities, randomized controlled trial

## Abstract

**Background:**

Nearly three-quarters of the estimated 4.9 million people with intellectual and developmental disabilities (IDDs) in the United States live with family caregivers, 25% of whom are aged older than 60 years. People with IDDs experience disproportionately high rates of mortality, morbidity, and acute care use, often due to a lack of accessible, tailored resources to support them throughout the lifespan. Few caregivers engage in long-term care (LTC) planning, often due to a lack of information and support.

**Objective:**

This protocol describes the design and methods of the Future Planning and Well-Being for Individuals with Intellectual Disabilities and Family Caregivers study, a randomized controlled trial evaluating the effectiveness of the Map Our Life web-based LTC planning tool compared to an attention control website on caregiver-reported burden, caregiver-reported well-being, and progress in and communication of future plans for the individual with an IDD.

**Methods:**

The Future Planning and Well-Being for Individuals with Intellectual Disabilities and Family Caregivers study is a national, multisite, randomized controlled clinical trial with a target enrollment of 1050 family caregivers of individuals with IDDs at 6 sites across the United States. Participants are randomly assigned (1:1) to either the Map Our Life web-based LTC planning tool plus enhanced usual care or an attention control group consisting of a Centers for Disease Control and Prevention–sponsored health promotion website for people with disabilities plus enhanced usual care. Primary outcomes, including caregiver-reported burden, caregiver-reported well-being, and LTC planning behaviors, are assessed at baseline and at 1, 6, and 18 months. Data will be analyzed using mixed-effects models to accommodate the repeated measures design.

**Results:**

This study was funded in July 2022, received initial Institutional Review Board approval in August 2023, and was registered on ClinicalTrials.gov in December 2023. Recruitment began in December 2023 and is scheduled to conclude in December 2025. Primary outcome analyses will commence immediately following the completion of final follow-up surveys.

**Conclusions:**

LTC planning is an important component of addressing health disparities among individuals with disabilities and their family caregivers. By focusing on using an innovative and accessible tool for LTC planning, the study addresses a critical gap in available resources with the potential to improve quality of life and reduce caregiver burden.

**Trial Registration:**

ClinicalTrials.gov NCT06065527; https://clinicaltrials.gov/study/NCT06065527

**International Registered Report Identifier (IRRID):**

DERR1-10.2196/77184

## Introduction

### Background

Of the estimated 4.9 million people with intellectual and developmental disabilities (IDDs) in the United States, nearly 75% individuals live with family caregivers, 25% of whom are aged older than 60 years [1]. As both individuals with IDDs and their caregivers age, they become increasingly susceptible to age-related health and functional decline, and caregivers face greater demands of caregiving [2-4]. Though caregivers worry about future care when they are no longer able to provide care [5,6], few families make concrete long-term care (LTC) plans or discuss future planning with their loved one with a disability. Main reasons may be, but are not limited to, not knowing where to start or finding the LTC planning process overwhelming [7]. This creates a pressing need for an LTC planning tool to help families become more informed about LTC needs and available supports.

IDDs are characterized by impairments in both intellectual and adaptive abilities originating in childhood, often associated with clinical comordities such as seizure disorders, may involve minimal or no understanding of verbal language, and often require enhanced or complete support throughout the lifespan [8-10]. Level of independence in adulthood varies depending on severity of the IDDs. Those with mild IDDs may achieve some signs of independence, while those with more severe IDDs often require long-term community supports for housing, occupational activities, and recreational activities [11,12]. Lack of concrete LTC plans may lead to acute crises and emotional trauma, introduce unexpected dilemmas for siblings or extended families, and at its worst, inappropriate or unwanted placement in nursing home settings, unsafe living conditions, or harm to individuals with IDDs [13-15]. Lack of comprehensive planning or planning without proper knowledge of how governmental support systems and legal systems operate could result in disabled individuals losing needed supports, being placed in restrictive environments, or potentially being stripped of their legal rights [15-17].

While LTC planning interventions exist for aging populations in general [18,19], few evidence-based interventions exist to support LTC knowledge, decision-making, and sharing of LTC plans specially tailored to meet the needs of individuals with IDDs and their family caregivers [20]. Plan Your Lifespan (PYL) is one such tool developed for adults in their fourth quarter of life [21,22]. The intervention, which was developed in accordance with the Preparation for Future Care Model, (1) introduces users to LTC related choices (care expectation), (2) assesses the unique caregiving needs of the care recipient (awareness), (3) educates the users on locally and nationally available home-based resources (information gathering), (4) makes choices about LTC preferences (decision-making), and (5) shares those choices with others (concrete planning). In a randomizad clinical trial, PYL demonstrated efficacy in assisting older adults with planning for the future and communciation of those plans to loved ones [23].

To address the lack of evidence-based interventions to support LTC planning for this population, research was conducted that identified a set of relavent domains of LTC planning, barriers and facilitiators in each domain, current LTC planning behaviors [23,24], and critical compnents to include in a web-based tool to support LTC planning [25]. The resulting product is an adapation of the PYL platform, called Map Our Life (MOL), that seeks to provide individuals with IDDs and family caregivers a unique and comprehensive tool by which to learn about future options in each identified domain and create an editable and sharable LTC plan for current and future use. It seeks to address current gaps in the literature around the existing evidence-based interventions to support LTC planning for this population and or their caregivers [26].

### Objectives

This study protocol outlines the rationale, design, methods, and statistical plan for a multisite randomized controlled trial, Future Planning and Well-Being for Individuals with Intellectual Disabilities and Family Caregivers. The study seeks to compare the effectiveness of MOL in combination with enhanced usual care (EUC) to an attention control (AC) website in combination with EUC on caregiver burden and well-being. The primary aim of the study is to compare caregiver quality of life outcomes by examining caregiver burden and self-efficacy scores at 1, 6 and 18 months. Our hypothesis is that family caregivers of those with IDDs in the MOL + EUC arm will have lower caregiver burden with time and higher self-efficacy scores at all points than those in the AC + EUC. Our secondary aim is to examine caregiver LTC knowledge and planning behavior and mediating and moderating effects on quality of life outcomes at 1, 6 and 18 months, comparing the effectiveness of EUC +MOL versus EUC + AC on these outcomes at 1, 6 and 18 months.

## Methods

### Study Design

This study is a multicenter, 2-arm, randomized controlled trial investigating the effectiveness of MOL + EUC compared to the AC + EUC. The goal is to recruit 1050 participants across 6 health systems in 5 states (Delaware, New York, Ohio, Pennsylvania, and Texas). Participants will be randomized in 1:1 ratio after consent has been obtained and baseline surveys are completed. Enrolled participants are expected to log into their assigned website at least once a week for a period of 3 months. Follow-up surveys will occur at 1, 6, and 18 months. Research coordinators refer participants to relevant disability support services in accordance with the EUC protocols developed, as described below. Findings of the study will be reported based on the CONSORT (Consolidated Standards for Reporting Trials) guidelines [27].

### Study Groups

#### Enhanced Usual Care

All study participants will receive enhanced usual care. Enhanced usual care will consist of regular case management services provided by disability service organizations in the participant’s geographic location. We will impose no constraints on usual care in the control or intervention group. Case management services will be administered through county- and state-based developmental disability service organizations that all participants are entitled to because of their qualifying diagnosis of IDDs. Upon enrollment in the trial, study staff will confirm a participant’s connection to their local disability service organization. In the event that the participant is not already connected to these services, study staff will provide a referral to connect participants to case management.

#### Map Our Life

In accordance with the Preparation for Future Care Model, Map Our Life (1) introduces users to LTC related choices (care expectation), (2) assesses the unique caregiving needs of the care recipient (awareness), (3) educates the users on locally and nationally available home-based resources (information gathering), (4) makes choices about LTC preferences (decision-making), and (5) shares those choices with others (concrete planning) [28]. Upon randomization to this arm, study staff will reach out to participants and provide log-in credentials and instructions on how to interact with the web tool. Participants will be asked to log into the site at least once per week for a month.

#### Attention Control

Caregivers in the attention control group will be referred to a website containing information from Disability and Health Information for Family Caregivers [29]. The content in the attention control is from a Centers for Disease Control and Prevention (CDC)-sponsored website that promotes healthy activities and behaviors targeting people with disabilities and their family caregivers. Additionally, the content leads users to CDC-sponsored “Caregiving” webpages, which assist families in developing care plans. Upon randomization to this arm, study staff will reach out to participants and provide log-in credentials. Participants will be asked to visit the site at least once per week for a month.

### Community Partner Engagement

This study is a community-engaged study, ensuring that integral feedback shapes the formation, dissemination, and application of research results and increases enrollment and retention. The Community Advisory Committee actively contributes to the project by sharing the study recruitment information through their networks, providing feedback on study questionnaires, intervention website content, and recruitment and retention materials. This collaborative approach ensures that the research remains aligned with community needs and promotes effective recruitment and retention strategies. To further engage community partners, quarterly Community Advisory Committee meetings are held, and newsletters are distributed to keep community partners informed and involved. Feedback from these meetings has been incorporated in recruitment materials and retention strategies, ensuring they are more accessible and culturally relevant to improve participant engagement.

### Eligibility Criteria

Eligible family caregivers must be at least 18 years of age and have a primary preferred language of English or Spanish. Family caregivers will be excluded if they do not have access to a computer, tablet, or smartphone that would allow engagement with the website or if the care recipient with IDDs is aged younger than 10 years at the time of recruitment.

### Recruitment and Screening

Potential participants are screened and enrolled by 1 of 6 health system sites (Christiana Care in Delaware, Northwell Health in New York, Children’s Hospital of Philadelphia in Pennsylvania, Cincinnati Children’s Hospital Medical Center in Ohio, Geisinger Health in Pennsylvania, and Baylor College of Medicine in Texas) by the sites’ respective investigative team. Each site will identify potentially eligible participants through a combination health system– and community-based recruitment. In health system–based recruitment, potentially eligible participants are identified based on encounters within participating health systems. Identification of potentially eligible participants in this setting occurs via health care provider direct referral, use of clinical registries, and review of ambulatory patient schedules. Community-based recruitment is spearheaded by community partners in conjunction with health system investigative teams and involves posting study information in community locations and sharing study information via approved social media outlets. Interested individuals are instructed to contact study staff to discuss study participation.

Once a potentially eligible participant is identified, study staff will meet with the potential participant in person or over the phone. Study staff describe the study and confirm eligibility before proceeding. At this time, presence of an IDD is confirmed. Regardless of recruitment method, all potential participants will be asked to self-report IDD diagnosis of the care recipient using the following validated questions from the National Survey of Children’s Health administered by the US Department of Health and Human Services [30]:

1. Has a doctor or other health care provider EVER told you that your child has Down Syndrome? (Yes/No)
2. Has a doctor or other health care provider EVER told you that your child has Cerebral Palsy? (Yes/No)
3. Has a doctor, other health care provider, or educator EVER told you that your child has Fetal Alcohol Spectrum Disorder (FAISD)? (Yes/No)
4. Has a doctor, other health care provider, or educator EVER told you that your child has Intellectual Disability (formerly known as Mental Retardation)? (Yes/No)
5. Has a doctor or other health care provider EVER told you that your child has Autism or Autism Spectrum Disorder (ASD)? Include diagnoses of Asperger’s Disorder or Pervasive Development Disorder (PDD). (Yes/No)

If any answer to any of the above questions is “yes”, the care recipient is considered to have an IDD. Study coordinators also will document whether the care recipient is a patient of one of the participating health system recruitment sites. If the care recipient is a patient at the recruitment site, the study coordinator will further confirm the presence of an IDD through chart review and health care provider confirmation of a specified list of International Classification of Diseases-10th Revision (ICD-10) codes.

**Figure 1:**
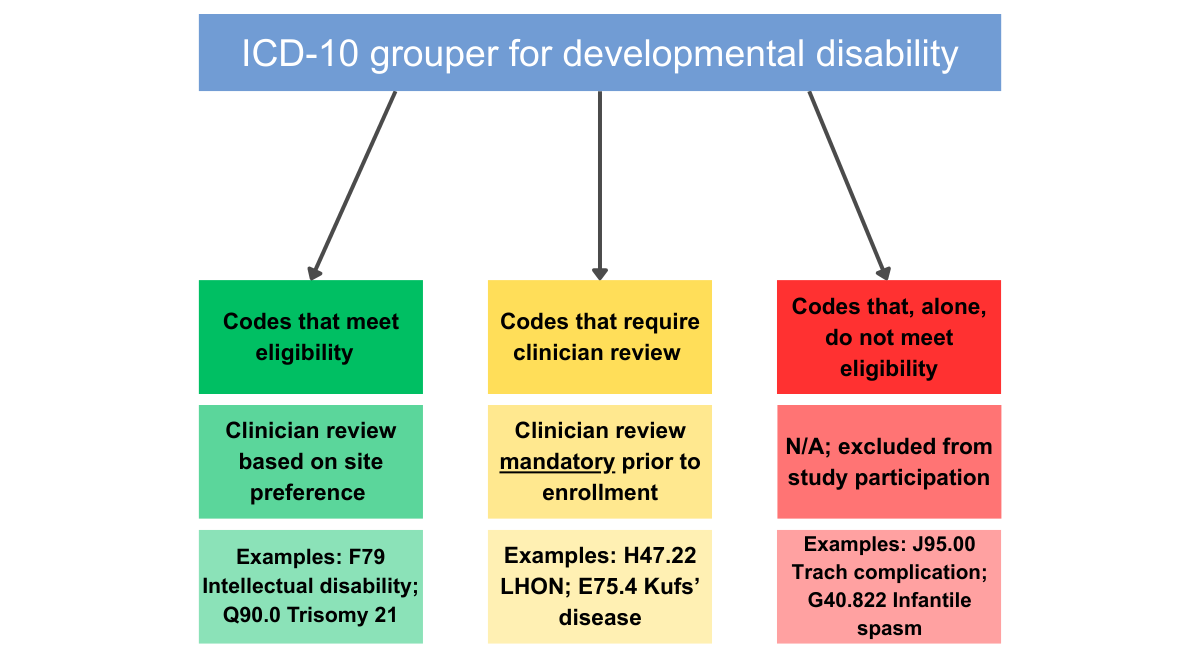
Identifying eligible patients through International Classification of Diseases-10th Revision (ICD-10) codes. The goal is to give the research staff a tool by which to identify potentially eligible patients for the study.

Expert consensus was used to tier ICD-10 codes within an existing health system grouper for developmental disability into three levels: (1) Tier 1 includes codes that meet eligibility where the research coordinator can approach patients without consulting a clinician. Tier 1 codes include diagnoses like Down syndrome (Q90.9), Cerebral Palsy (G80.0), Fetal Alcohol Spectrum Disorder (Q86.0), Intellectual Disability (F79), and Autism Spectrum Disorder (F84.0). (2) Tier 2 codes require clinician review for confirmation of eligibility and include diagnoses like focal motor seizure (G40.109), Spina Bifida (Q05.9), and pervasive developmental disorder (F84.9). These are diagnoses for which we expect at least 50% of impacted individuals to experience an IDD. (3) Tier 3 codes alone do not meet eligibility. Thus, a patient with a tier 3 code and no other diagnosis is excluded (Figure 1). If the self-reported information conflicts with what is in the care recipient’s chart, for eligibility purposes, the information provided by the caregiver will outweigh information gathered in the chart.

### Assent and Consent

#### Caregiver Consent

Once screening is complete and eligibility is confirmed, as described above, eligible caregivers will be approached by research staff to review study procedures and provide consent for participation. This process may occur in person or over the phone. Electronic consents will be obtained via REDCap (Research Electronic Data Capture). During this process, the study will be described and the consent form will be reviewed with the participant.

The consent script will be available in both English and Spanish to ensure all individuals understand the nature and requirements of the study. Communication with Spanish-speaking participants will be conducted through a medically certified interpreter, and information about the interpreter will be documented in REDCap. Once finished, the participant will provide an e-signature via REDCap. Participants will be emailed a copy of the e-consent form. Waiting time between informing prospective participants and obtaining consent will depend on the mode used to inform participants about the study.

#### Care Recipient Consent

Once a caregiver agrees to participate in the study, the ability of the care recipient to assent or consent will be determined. If a care recipient is aged older than 18 years and cognitively able to provide consent independently, they will be asked to complete survey measures in addition to their caregiver. Care recipients who are unable to independently provide consent will not be asked to complete survey measures. Their caregiver will complete care recipient–designated survey measures as a proxy respondent. The research team will take extra care to ensure that participants with disabilities understand the study goals and what is being asked of them as study participants.

### Randomization

After caregiver participants complete the baseline surveys, they are randomly assigned to 1 of 2 groups: the web-based long-term care planning tool, Map Our Life, with enhanced usual care (MOL + EUC) or an attention control group, a CDC-sponsored health promotion website for people with disabilities, with enhanced usual care (AC + EUC). Participants are randomized in a 1:1 concealed allocation procedure that uses a permuted block design with randomly varying block sizes of 2 and 4. Randomization will occur via REDCap’s randomization module. Both groups will be provided with a short tutorial video and prompted to log into their assigned website once a week for a period of 3 months.

### Follow-Up Surveys

Follow-up surveys are completed at the following intervals: at 1 month, 6 months, and 18 months after randomization. These surveys can be completed online, over the phone, or in person. The surveys ask demographic questions about the caregiver and care recipient as well as questions about caregiver and care recipient quality of life (using the Zarit Burden Interview [ZBI] [31,32], Revised Scale for Caregiving Self-Efficacy [33-35], PROMIS Global Health [36-38], and World Health Organization Quality of Life Disability Module [39]) and health behaviors that relate to long-term care planning (using Planning Behavior and Communication of Plans [23,40,41], COPE [42,43], and the National Core Indicators Family Survey – Information and Planning [44-46]). A list of the different tools, their administration time point, and the typical amount of time required for completion can be found in Table 1. Participants can receive up to $175 over the 18-month study period for survey completion.

**Table 1:** Patient-reported outcome measures.

Measure	Number of items	Time point (month)	Person responsible
Caregiver Zarit Burden Interview [31,32]	22	0, 1, 6, 18	Caregiver
Revised Scale for Caregiving Self-Efficacy [33-35]	5	0,1,6,18	Caregiver
World Health Organization Quality of Life Module [39]	13	0,6	Caregiver, care recipient
Planning Behavior and Communication of Plans [23,40,41]	21	0,1,6,18	Caregiver
COPE: Use of instrumental social support subscale, active coping subscale, planning subscale, denial [42,43]	16	0,1,6,18	Caregiver
National Core Indicators Family Survey – Information and Planning: Services and Supports Received Subdomains [44-46]	20	0,1,6,18	Caregiver

### Data Collection

In addition to data collected as part of the various questionnaires, participants provide basic demographic information about themselves and the care recipient (caregiver: age, gender, race, ethnicity, preferred language, education, relationship to care recipient, marital status, employment, and income; care recipient: age, gender, race, housing arrangement, employment, and Medicaid waiver participation). Additionally, unpaid care time (hours per week) and the number of care recipients in a household are recorded. All data collection occurs in a Health Insurance Portability and Accountability Act (HIPAA)-compliant REDCap database overseen by the coordinating institution.

### Statistical Analysis

The study will follow the intention-to-treat principle, analyzing participants in the arm they were randomly assigned to. Data and measures will be summarized according to treatment arm using means and SDs, or medians and IQRs for continuous variables, as appropriate. Frequencies and percentages will be reported for categorical variables. No inferential comparisons of the 2 arms will be carried out. For aim 1, a 2-sample *t* test will be used to compare the 2 treatment arms at the 12-month follow-up. Ninety-five percent CIs for the mean difference between the 2 arms will be constructed. If the assumption of normality does not hold, the Mann-Whitney test will be used instead. As suggested by International Council for Harmonisation (ICH) E-9 guidelines, a supporting analysis will be carried out using analysis of covariance (ANCOVA) to compare the 2 treatment arms, adjusting for the stratification variables (institution, age of the person with an IDD, and Waisman score). A secondary analysis will be carried out using ANCOVA to compare the 2 treatment arms, adjusting for the stratification variables, baseline outcome value, and the prespecified covariates: race (White, non-Hispanic compared to American Indian or Alaska Native, Asian, Black or African American, Native Hawaiian or Pacific Islander, Hispanic or Latino, or another race or ethnicity) and language (English and Spanish). The approaches used for analyzing the primary outcome will also be used for all continuous secondary outcomes. In addition, longitudinal analysis of the primary and secondary outcomes (ZBI, caregiver self-efficacy, and community participation) will be carried out using generalized linear mixed models for repeated measures, adjusting for stratification variables as well as the additional prespecified covariates race and language.

Power calculations were performed for 2 different sample sizes (400 and 500 participants per treatment arm) under several scenarios. We will use absolute change in ZBI score at the 6-month follow-up as our primary outcome of interest. Based on a similar study by González-Fraile et al [47], the SD of the absolute change in total ZBI was 13.94 in the treatment arm and 10.02 in the control arm. Accordingly, we considered 3 values for SD, namely, 10.02, 13.94, and the variance-based average of 11.99. Based on clinical experience, assuming that the average change in the control arm is approximately 0 and the average change in the treatment arm will be 3 [48], the resulting effect size is 0.25 to 0.3. depending on which value of SD holds. We further assume that our projected sample size is 525 participants per arm. This would yield power of between 93.3% and 99.8% for the 3 possible SD values. If 20% of the sample is lost to follow-up at 6 months and the projected sample size is 420 per arm, the resulting power values range from 87.6% to 99.1% for the 3 SD values (2-tailed 2-sample *t* test, α=.05).

### Ethical Considerations

The study is registered on ClinicalTrials.gov (NCT06065527). This protocol was reviewed and approved by the Feinstein Institutes for Medical Research/Northwell Health (23-0381) institutional review board (IRB). This study uses a single IRB to promote consistency across sites. As described above, study staff clearly describe study procedures to family caregiver participants prior to asking them to provide consent for participation. A rigorous process is used to determine the capacity for individuals with IDDs to provide consent or assent. Data in this study are not anonymized; however, protections are in place to safeguard participant information, including the use of HIPAA-compliant data capture systems and restricted access to participant data based on study role. Participants are compensated for survey completion and can earn up to $175 for study participation. This study is considered minimal risk. Mitigation of primary concerns, breach of confidentiality, and participant discomfort include data deidentification methods, password-protected storage, and voluntary participation from participants.

## Results

This study was funded in July 2022 from the Patient-Centered Outcomes Research Institute (grant AD-2021C3-24941), received initial IRB approval in August 2023, and was registered on ClinicalTrials.gov in December 2023 (NCT06065527). Participant recruitment began in December 2023 and is scheduled to conclude in December 2025. The results of these analyses will be reported in a separate manuscript.

## Discussion

This study is designed to contribute significant knowledge about the potential benefits of a web-based LTC planning tool for individuals with IDDs and for their caregivers. The results will also assist individuals with IDDs and their caregivers, in collaboration with their health care providers, in developing and communicating LTC plans. The website used by the intervention group may be implemented in different care settings and for other diseases and thus generate generalizable knowledge. Furthermore, it could serve as a model for developing similar interventions for other populations with complex long-term care needs.

A defining strength of this study is its comprehensive and ethically driven approach to participant inclusion, which includes tiered screening, legal guardian consent, and care recipient assent or consent processes. These methods ensure that all participants are appropriately assessed and included based on their unique needs and abilities while maintaining a high standard of ethical research practices. This process not only safeguards the rights of individuals with IDDs but also provides a replicable framework for future studies involving populations with varying levels of cognitive ability. Moreover, the integration of stakeholder feedback throughout the study ensures the intervention aligns with community needs, thereby increasing its acceptability and retention. This collaborative approach strengthens the study’s relevance and potential for real-world application in underserved populations in research.

One limitation of this study is that caregiver burden and LTC planning behaviors are assessed through self-reported data that could introduce bias. Second, while the study includes participants from diverse sites across the United States, our study only supports Spanish and English languages, underrepresenting certain populations and potentially limiting the generalizability of findings. Additionally, the study’s design requires internet access, potentially excluding caregivers who do not have access to or are unable to use the specified technology.

The proposed protocol offers a comprehensive framework for evaluating a web-based LTC planning intervention for caregivers of individuals with IDDs. By integrating innovative methodologies for screening, legal guardian consent, and care recipient assent or consent, the study addresses ethical complexities while ensuring inclusivity and rigor. If successful, the intervention could transform LTC planning, reducing caregiver burden and improving outcomes for individuals with IDDs. The findings may inform future research, clinical practice, and policy development, advancing care and support for this underserved population.

## Abbreviations

AC: attention control
ANCOVA: analysis of covariance
CDC: Centers for Disease Control and Prevention
CONSORT: Consolidated Standards for Reporting Trials
EUC: enhanced usual care
HIPAA: Health Insurance Portability and Accountability Act
ICD-10: International Classification of Diseases-10th Revision
ICH: International Council for Harmonisation
IDD: intellectual and developmental disabilities
IRB: Institutional Review Board
LTC: long-term care
MOL: Map Our Life
REDCap: Research Electronic Data Capture
ZBI: Zarit Burden Interview

## Data Availability

Data sharing is not applicable to this article as no datasets were generated or analyzed during this study. At the end of the study, the datasets generated or analyzed during this study will be available from the corresponding author on reasonable request.
